# Our Cosmic Insignificance

**DOI:** 10.1111/nous.12030

**Published:** 2013-06-04

**Authors:** Guy Kahane

**Affiliations:** Oxford University

## Abstract

The universe that surrounds us is vast, and we are so very small. When we reflect on the vastness of the universe, our humdrum cosmic location, and the inevitable future demise of humanity, our lives can seem utterly insignificant. Many philosophers assume that such worries about our significance reflect a banal metaethical confusion. They dismiss the very idea of cosmic significance. This, I argue, is a mistake. Worries about cosmic insignificance do not express metaethical worries about objectivity or nihilism, and we can make good sense of the idea of cosmic significance and its absence. It is also possible to explain why the vastness of the universe can make us feel insignificant. This impression does turn out to be mistaken, but not for the reasons typically assumed. In fact, we might be of immense cosmic significance—though we cannot, at this point, tell whether this is the case.

As we complacently go about our little Earthly affairs, we barely notice the black backdrop of the night sky. Even when we do, we usually see the starry skies as no more than a pleasant twinkling decoration. In one sense, what we see is not very different than what Neolithic or Medieval people saw when they looked up. But we inhabit a different universe.

We can repeat the figures scientists tell us, though, mercifully, we do not entirely comprehend them. Scientists tell us, for example, that the universe is more than 13 billion years old, and that the diameter of the part of the universe that we are able to observe is at least 93 billion light years, though for all we know the universe might be infinite in volume. Our own planet circles a star that is located around two thirds of the way out of the centre of the modest Milky Way galaxy, which contains 100–400 billion stars. This isn't very much: according to the latest calculations, the observable universe contains around 300 sextillion stars.^1^ These figures are, well, astronomical, but they are also misleading. On the whole, our universe is almost entirely empty, an unending cold intergalactic night.

We float in this immense cosmos, Carl Sagan writes, “like a mote of dust in the morning sky.”^2^ Stephen Hawking delivers the news more bluntly. We are, he says, “just a chemical scum on a moderate-sized planet, orbiting round a very average star in the outer suburb of one among a hundred billion galaxies.”^3^

The universe is immense, and we are so very tiny. When we contemplate the vastness of the universe we inhabit, our humdrum location, and our inevitable future doom when the sun implodes, or later on, in the heat death of the universe, human life can seem utterly insignificant.

This sense of cosmic insignificance is not uncommon. Pascal famously wrote,

When I consider the short duration of my life, swallowed up in an eternity before and after, the little space I fill engulfed in the infinite immensity of spaces whereof I know nothing, and which know nothing of me, I am terrified. The eternal silence of these infinite spaces frightens me…^4^

One of Joseph Conrad's characters describes

one of those dewy, clear, starry nights, oppressing our spirit, crushing our pride, by the brilliant evidence of the awful loneliness, of the hopeless obscure insignificance of our globe lost in the splendid revelation of a glittering, soulless universe. I hate such skies.^5^

The young Bertrand Russell, a close friend of Conrad, bitterly referred to the Earth as “the petty planet on which our bodies impotently craw.” Russell wrote that:

Brief and powerless is Man's life; on him and all his race the slow, sure doom falls pitiless and dark. Blind to good and evil, reckless of destruction, omnipotent matter rolls on its relentless way… 6

This is why Russell thought that, in the absence of God, we must build our lives on “a foundation of unyielding despair.”

Such quotations could be easily multiplied—we find similar remarks, for example, in John Donne, Voltaire, Schopenhauer, Byron, Tolstoy, Chesterton, Camus, and, in recent philosophy, in Thomas Nagel, Harry Frankfurt, Ronald Dworkin, and Susan Wolf.^7^

Many take our insignificance to be an obvious, undeniable truth. But are we really cosmically insignificant? Why should we be insignificant, just because the universe is so vast? Over forty years ago, Thomas Nagel remarked that “[r]eflection on our minuteness and brevity appears to be intimately connected with the sense that life is meaningless; but it is not clear what the connection is.”^8^ This connection remains unclear. And the same could be said about the supposed significance of our humdrum location, or the inevitable future extinction of human life.

These questions about our significance do not receive much philosophical attention. Many contemporary philosophers, if they even notice such questions, consider them an embarrassment to be avoided, the product of a simple metaethical muddle. The very idea of cosmic significance is ridiculed.

We shall see that this gets it all wrong. The experience of cosmic insignificance has little to do with metaethics. We can make good enough sense of the idea of cosmic significance and its absence. And we can see why our minuteness compared to the vastness of the universe can make people feel insignificant. This impression does turn out to be mistaken, but not for the reasons typically assumed. In fact, it turns out that we might be of immense cosmic significance, even universally central, in the only sense that matters. But as I will later explain, whether or not we are cosmically significant is still an open question. There is also, however, an important sense on which we clearly *are* insignificant. But again, this is so for different reasons than those usually assumed.

## Insignificance and Metaethics

Bernard Williams is a good example of contemporary responses to worries about cosmic significance. Williams thinks that such worries express a banal metaethical confusion, the failure to distinguish between

thinking that our activities fail some test of cosmic significance, and… recognizing that there is no such test. If there is no such thing as the cosmic point of view, if the idea of absolute importance in the scheme of things is an illusion, a relic of a world not yet thoroughly disenchanted, then there is no other point of view except ours in which our activities can have or lack a significance.^9^

To worry about our cosmic insignificance is thus confused. Not because we actually possess cosmic significance. Rather the very idea of such significance is incoherent.

Williams assumes here that there is no test of cosmic significance, a notion he identifies with ‘absolute significance’—in other words, with what is better called objective value. Now it would indeed be a mistake to infer, *just* from the claim that there is no objective value, that *nothing* has value. But it's *equally* mistaken to infer, as Williams does, from the fact that some things are valuable *to* us, that these things would be truly valuable whether or not objective value exists. Both inferences lack a crucial conceptual premise. If to be valuable just is to be the object of our concern, then Williams is perhaps right that it doesn't matter whether objective value exists. But if to be valuable is to be *objectively* valuable, then, as Mackie pointed out, if objective value doesn't exist, then it *does* follow that nihilism is true and nothing matters, whether or not we care about some things.^10^ The supposed inexistence of objective value leaves it open which of these conceptual claims is correct. This is a familiar point.

Williams presents the rejection of objective value as a consequence of naturalism, and of the discovery that God does not exist. In fact, not long before the passage quoted above, Williams writes that if God had existed, and found us significant, then we *would* be cosmically significant, and ‘valuable tout court’.

Now objective value might be incompatible with metaphysical naturalism, but, for familiar reasons, it is implausible to think that it is ruled out simply because God doesn't exist. And it's anyway hard to see how the size of the universe would bear on the question of God's existence. If God does in fact exist, this won't show the universe to be any *smaller* than science now tells us it is.

Although worries about objective value arise most acutely once theism is abandoned, their real source isn't atheism, but naturalism. Their real source isn't the inexistence of a divine commander but the exclusion, implied by naturalism, of the supernatural, queer and spooky. It is still under dispute, however, whether accepting the picture of the universe offered by natural science also commits us to denying the existence of objective value, whether understood in naturalist or even non-naturalist terms.

But we can set aside these large metaethical questions. The main point is that even if there is a tension between naturalism and objective value, it is due to the way naturalism is supposed to exclude certain entities, properties and facts. What is again unclear is what the size or age of the universe has got to do with any of that.

If naturalism indeed ruled out objective value, it would rule out objective value even if the naturalist universe was the size of a matchbox, or came into existence five minutes ago. It would rule it out even if we were located precisely at the centre of the universe. Conversely, if objective value exists, whether in some Platonic realm or in some other sense (naturalist or not), then it would exist regardless of the size and age of the universe. It would exist even if the universe was infinitely vast, infinitely old, and absolutely indifferent and silent.

It is obvious, then, that the size of the universe, and our tiny dimensions, have no metaethical significance. They lend no support to nihilism, nor do they tell us anything about the existence of objective value. We can conclude that if our sense of cosmic insignificance has *anything* to do with the size and age of the universe, it has little to do with the tension that some metaethicists see between naturalism and objective value.^11^

There is a further problem with the metaethical reading of insignificance. If the sense of insignificance was an expression of metaethical nihilism, then it could hardly be cause for despair. Nihilism says that nothing matters. This, however, would include the truth of nihilism: if nothing matters, this *also* doesn't matter. If *anything* is a muddle, it is to respond with despair to the (supposed) truth of nihilism.

## The Argument from Intrinsic Value

It is thus implausible that these metaethical issues are really at the heart of the experience of insignificance. But if this experience doesn't express a metaethical worry, then it must express a *substantive* worry—not a metaethical challenge to our evaluative scheme, but a first-order move *within* it.^12^

In its simplest form, the idea would be that we possess no, or little value, *because* we are so small and the universe so vast.

This suggestion, however, also makes little sense. If something possesses intrinsic value, value in virtue of its intrinsic properties, then how could the size of the universe, or indeed *anything* about the surrounding universe, affect its value in any way?^13^

Our intrinsic value is also independent of whether we are located at the centre of the universe or on its margins, or on whether and when humanity will one day go extinct. Consider for example pain. If pain is bad in virtue of what it's like, then a given instance of pain would be just as bad—and bad to exactly the same degree—whether it occurs in a Gulliver or in a Lilliputian, whether it occurs in a tiny or in a vast or even infinite universe, whether it occurs in the centre or the corner, to the left or to the right.^14^

Now not all things we value in themselves are also intrinsically valuable. Some things have value that is *final* but *extrinsic*^15^—final value *can* depend on extrinsic properties, including the fact that one is so small and the universe so vast. So the above argument might be too quick.

It is true that the final value of something might in principle depend on the size of the universe, or our comparative size, or, really, on *any* fact or relation. This, however, is no more than an uninteresting logical possibility. To start with, the paradigm cases of final value, such as the badness of pain, or the moral significance of persons, are also cases of intrinsic value. As we saw, if pain is intrinsically bad, then it is bad even if the universe is infinite, even if time goes on without limit.

But even if some central instances of value were of final value that is extrinsic, it is still obscure how we are to cash out the logical possibility that this value is diminished by the size of the universe. It's often argued that spatial location and distance are morally irrelevant properties. Cosmological facts about the dimensions of the universe seem similarly axiologically irrelevant. If something possesses value, why should it matter whether that thing is located in a tiny cabinet or in a vast chamber?

It's generally absurd to think that size matters in itself. As Russell wrote in a later piece, “there is no reason to worship mere size… Sir Isaac Newton was very much smaller than a hippopotamus…”^16^ To be sure, we sometime use size as a metaphor, or as an expressive vehicle, when we try to represent what we take to have great value. It is hard to think of a religion that worships an infinitesimally tiny deity, and monuments typically aspire to, well, be monumental. But this is merely because the large is more psychologically impressive than the small, and because it's a greater achievement to build a pyramid or cathedral than an equivalent miniature. Still: few would prefer an immense dung heap to a diamond.

## Significance Is Not the Same as Value

We could conclude that worries about our cosmic insignificance are confused. But it would be better to conclude that they don't express metaethical worries about the objectivity of value, or the first-order worry that we posses no value.

Claims about significance are indeed first order-claims, and are related to claims about value. But it's implausible that they are *no more* than claims about value. For something to be significant, it needs to possess (or at least bring about) some value. But it is clear that possessing some value isn't sufficient for being significant. For something to be significant, it needs to be *important*, to *make a real difference*. When we describe something as insignificant, we rarely mean to deny that it possesses *any* value. We are just denying its importance.^17^

I will not try to offer a complete analysis of importance.^18^ It is enough to say that for something to be important, it needs to possess, or bring about, enough value to make a difference—and that if something *is* important, then it merits our attention and concern. Conversely, if something is *insignificant*, then it's *not* noteworthy, not important enough to care about.

We cannot be concerned about everything that has value. This is in part because that would be impossible. Just think of all the vast amount of suffering happening in the world right now; pain is bad, but we couldn't possibly pay attention to, let alone care about, each and every instance of pain on the planet. But the gap between value and what we have reason to care about isn't due only to our profound cognitive and emotional limits. Seen alongside the horrific (or the wonderful), many things become simply insignificant—worthy of no attention at all. If you witness a terrible tragedy, it would be inappropriate to obsess about a stain on your suit. After spending a week attending to soldiers injured and disfigured in the civil war, Whitman wrote to his mother that “… really nothing we call trouble seems worth talking about.”^19^

Because, unlike value, significance is relative to point of view, it can vary in this way even as value stays fixed. What attention and concern is merited by something is a function not only of its own value, but also of what else of value is in view. The intrinsic value of something cannot be changed by its surrounding. But its significance can. And when it is seen in an ever broader perspective, the significance of a thing can decline until it simply no longer matters.^20^

It is thus a mistake to confuse worries about our significance with worries about whether we, or anything at all, possess value. To be sure, if nihilism is true, then nothing is really significant—whether on the cosmic scale *or on any other scale*. But to worry about our significance in *that* context is a bit like complaining you didn't win the lottery when you don't even have a ticket. More importantly, even if nihilism is false, and our lives *are* valuable it still wouldn't follow that we are significant. We might still be utterly unimportant, our existence might make no real difference.^21^

## Cosmic Significance

We can tell how significant something is by asking what attention it deserves, all things considered. We can consider the significance of things at different scales: around now, in Logic Lane; in London, on September 1666; in the Holocene, on Earth… The *cosmic* significance of a thing simply refers to its importance, and thus to what attention is deserves, when it is viewed from an impartial standpoint, all things considered—*when literally all things are considered*.

The cosmic standpoint is the most encompassing evaluative perspective, the standpoint that considers everything, everywhere, without exception. It isn't the peculiar perspective *of* the cosmos, whatever that might mean. It is not so much a view from *nowhere* as a view of *everywhere*.

The cosmic standpoint is often conflated with the notion of objective value, or even taken to be a kind of metaethical device.^22^ This is a mistake, a confusion of a form of assessment that is distinctively unqualified in its *scope*, with a very different metaethical claim about the *source* of value. The cosmic standpoint does transcend our parochial, anthropocentric perspective. But it makes no claim about the ‘fabric of the world’.

It is true that on many antirealist views, value and thus significance would ultimately be explained by reference to our subjective concerns, themselves the product of a contingent terrestrial process. Cosmic significance may ultimately have an Earthly source.^23^ This does sound odd.

But if such an anthropocentric view is correct, we can still talk about value, and, presumably, of significance, on different scales. It is hard to see what stops us from also talking about significance on the grandest, cosmic scale. If you find it odd to say that cosmic significance ultimately arises from our own subjective attitudes, you should find it equally odd that the badness of the agony of a dying prehistoric mammal is due to our own attitudes millions of years later. If there is a problem here, it is for these forms of antirealism, not for their compatibility with the cosmic standpoint.

It is common for non-philosophers to confuse antirealism with relativism. Value might have its source in our subjective attitudes without this implying that things possess value only relative to Westerners, or Easterners, or Holy Romans, let alone that we should be especially tolerant of others cultures, however outrageous. But it's equally a mistake to think of antirealism as implying that value is in some way terrestrial—as if the value of things begins to fade away as we leave the Earth's atmosphere. (Or is it wrong for antirealists to find the starry skies beautiful?)

Many antirealists about value are also antirealists about other things, such as mathematics. But it would be absurd to think that if we are antirealists about maths, then it's somehow wrong for us to try to calculate, say, the age or size of the universe… So why should the idea of cosmic significance present any special problem?

It is common, almost instinctive, to respond to worries about our cosmic significance by rejecting objective value, by insisting that, as Williams puts it, “there is no other point of view except ours in which our activities can have or lack a significance.”^24^ We can now see that this response is off mark. We can ask, and worry, about our cosmic significance even if our own point of view is the source of all value. And if antirealism is true, this in no way guarantees that the answer will be favourable.

This point also offers an answer to the objection that nothing is *simply* important—that things are always important, or matter, *to someone*. To the extent that this objection is an expression of an antirealist understanding of value, then I believe I have already answered it: there is actually no tension between antirealism and the idea of cosmic significance, even if on some forms of antirealism value is ultimately to be explained by reference to our subjective concerns. And even on these views, we can make mistakes about value: what is valuable isn't simply what we happen to value. In a similar way, what is significant couldn't just be what we *take* to be significant, or actually attend to or care about; it's a truism that the profoundly trivial often attracts obsessive attention.

The objection, however, could also take a different form. It might be argued instead that things are never important tout court, but only relative *to* some person, value or aim. Now importance can certainly take this relative form. Some things are important *for* us—important with respect to us and our good, and thus deserve special attention from our personal standpoint (though they needn't in fact receive that attention—they may not be actually important *to* us). The question is whether things can also be important, and thus deserve attention, from an impartial standpoint. All that such a notion of general importance requires is that it be possible to compare the overall value that different things possess and affect. Although such comparisons are difficult, and often quite imprecise, I don't see that they present great conceptual difficulties. We make such comparisons, and ascribe general importance to things, all the time, even if we rarely consider importance on a genuinely terrestrial, let alone cosmic, scale.^25^ Notice also that the notion of cosmic significance is perfectly compatible with the view that things are good and bad only *for* persons, and other sentient beings. On such a view, we could still compare the overall difference in value, and effect on value, that things make.^26^

## Our Supposed Cosmic Insignificance

We can now finally explain—though not ultimately vindicate—the sense of insignificance we experience in the face of a vast universe.

Things tend to dramatically lose their significance as the context broadens. A tragic incident that deserves many pages in the Didcot Gazette may merit only a footnote in the History of Oxfordshire, and not a single word of the Annals of England… But then, how much of what makes up the Annals of England would make it to the Final History of the World?

When we consider ourselves as a mere dot in a vast universe, when we consider ourselves in light of everything there is, nothing human seems to matter. Even the worst human tragedy may seem to deserve no cosmic concern. After all, we are fighting for attention with an incredibly vast totality. How could this tiny speck of dust deserve even a fraction of attention, from that universal point of view?

This is the image that is evoked when, for example, Simon Blackburn writes that “to a witness with the whole of space and time in its view, nothing on the human scale will have meaning”.^27^ The bigger the picture we survey, the smaller the part of any point within it, and the less attention it can get… When we try to imagine a viewpoint encompassing the entire universe, humanity and its concerns seem to get completely swallowed up in the void.^28^

This, then, is how the size of the universe might seem to matter.

## Are We Really Invisible from the Cosmic Standpoint?

It is natural enough to imagine the cosmic view as a something similar to those deeply dark images of outer space that our telescopes produce. But the cosmic standpoint isn't literally about what one would see if one was located in some special cosmic observatory, and peered into a super-powerful telescope.

It is true that if you see someone in pain from far away, the emotional hold of her suffering quickly diminishes. For this to happen you do not need to ‘see’ her from the distance of the Andromeda galaxy. It's enough to look at her from somewhere slightly high up—not from the cosmic viewpoint, but from a modest *bird's* eye view. But this is irrelevant. The cosmic point of view encompasses an immensity, but this isn't the same as seeing things from a great *distance*.

It is a mistake to take the visual metaphor too literally.^29^ When we adopt the cosmic standpoint, it is not as if we become *more* ignorant, know even less than we do now. But if we still know about the lives and agony of terrestrial beings, and if these possess value, then this value should still be registered.

It thus makes no sense to worry that we, and our value, are literally invisible from the cosmic standpoint. The only question is whether, seen from this all encompassing standpoint, we and our value really *matter*. The worry remains that we must compete for attention with everything else in this vast universe. If the cosmic response is divided between everything, any response to even the worst terrestrial horror would be infinitesimal. We are, after all, less than drops in an immense ocean.

But this worry is also confused. If you lie in agony, surrounded by row after row of fellow sufferers, then your own particular pain disappears into this multitude. But if you are a single sufferer, in what way could it matter whether you cry out in a cramped closet or in a vast empty hall? Of course it might be harder to *hear* you from the edge of that hall—even more so, we might suppose, from the edge of the universe—but that is merely an uninteresting epistemic point.

What is most striking about the immensity that surrounds us is that it is very nearly empty. And even when it is not mere empty space, it very nearly contains nothing of value—in itself it in no way matters if a lifeless gas giant explodes, or if a cosmic cloud is devoured by a black hole.^30^

## What Would Really Decide Our Cosmic Significance

When we are impressed by our tiny size, by the vastness of the space that envelopes us, and conclude that we must be very unimportant, this may be because we forget to consider just how empty this immensity is. An observer might take a very long time to find us in this immensity, but besides us, he might find in it little or nothing to care about.

That the naturalist universe is virtually empty of value is hard to dispute. It is also hard to dispute that we are one glimmer of value in the darkness. The real, the big question is whether we are the only such glimmer.

Over the centuries, many have thought it absurd to think that we are the only ones. For example, Anaxagoras, Epicurus, Lucretius, and, later, Giordano Bruno, Huygens and Kepler were all confident that the universe is teeming with life.^31^ Kant was willing to bet everything he had on the existence of intelligent life on other planets.^32^ And we now know that there is a vast multitude of Earth-like planets even in our own little galaxy.^33^ It is also the case, however, that we do not yet possess even a shred of evidence that life, let alone intelligent life, exists outside of our planet. At least for now, it remains possible that we are the only ones.^34^

Arthur C. Clarke famously said that “There are two possibilities: either we are alone in the universe, or we are not. Both are equally terrifying.” Reflections on our cosmic significance reveal one important way in which this is true.

## If We Are Alone

We can consider first what would follow if we are alone in the universe.

If we are alone, then we, and other terrestrial sentient beings, might be the only thing that possesses intrinsic value in the entire cosmos. If so, then we are the only thing that deserves an evaluative response, the *only* thing that matters, even from the cosmic perspective. We are the only thing of value, and thus also *the only thing that is cosmically significant*.

If we are alone, then we humans also possess the *most* value in the cosmos, and also arguably the greatest *part* of total cosmic value (compared to other terrestrial sentient beings). And what we do, and what happens to us, is what determines the overall value *of* the cosmos. If humanity fails, if we create hell on Earth, then we make the universe itself bad.^35^ And if (or rather when) life on Earth become extinct, this might be the end of value in the universe.

Williams and many others are thus mistaken. *If* we are alone, then, pathetically small as we may be, we are also of *immense* cosmic significance.

Oscar Wilde famously quipped that “We are all in the gutter, but some of us are looking at the stars.”^36^ Now we may be in the dumps, and there's no denying the beauty of the stars, but ultimately, in this vast universe, our gutter is perhaps still the only thing really worth looking at.^37^

## The Thinking Reed

In another famous passage, Pascal wrote that

Man is but a reed, the most feeble thing in nature; but he is a thinking reed… if the universe were to crush him, man would still be more noble than that which killed him, because he knows that he dies and the advantage which the universe has over him; the universe knows nothing of this.^38^

Writing with a lighter touch, Frank Ramsey similarly remarks that

Where I seem to differ from some of my friends is in attaching little importance to physical size. I don't feel the least humble before the vastness of the heavens. The stars may be large but they cannot think or love; and these are qualities which impress me far more than size does. I take no credit for weighing nearly seventeen stone.^39^

Pascal and Ramsey seem to be gesturing at the point that despite the vastness of the universe, its empty spaces, and the numerous scattered stars, possess no value, whereas we, small as we are, do possess value in virtue of our capacity to think and love.^40^ As we have seen, however, this in itself won't help: the mere fact that we possess such value isn't sufficient to establish our significance. Our significance would rather follow from the *conjunction* of (and contrast between) our value *and* the fact—if it is a fact—that we are surrounded by a vast emptiness.^41^

## Ramsey's Picture of the World

Ramsey also wrote that

[m]y picture of the world is drawn in perspective, and not like a model to scale. The foreground is occupied by human beings and the stars are all as small as threepenny bits.^42^

On this picture, we are at the centre, and everything else is just there as background colour, confetti decoration for the terrestrial stage.

Ramsey's picture of the world is avowedly anthropocentric. It presents things as they seem from the human standpoint. This is the view that Williams also recommends. But in endorsing this picture, Williams ignores a massive inconvenience. Ramsey's picture is explicitly antirealist. Not antirealist just about value, the way Williams is, but about the *universe itself*. As Ramsey writes immediately afterwards: “I don't really believe in astronomy, except as a complicated description of part of the course of human and possibly animal sensation.”

One implication of the argument so far is that Ramsey's picture *might nevertheless be correct*. It might be the correct view of things *from the cosmic standpoint*—on condition that we are the only ones. For this picture might be also understood not as an expression of antirealism, or a representation of our parochial terrestrial take on things, but as a view of the *entire universe*, when things are represented in proportion to their significance, a bit like the way the human body is represented in the brain, not to scale, but in proportion to sensory density.

If we are the only ones then, ironically, the cosmic standpoint ends up largely overlapping with our current terrestrial concerns, focused almost exclusively on sentient and intelligent life on the surface of a tiny planet surrounding a humdrum star in an (otherwise) unremarkable galaxy.

## Our Extrinsic Value

We saw earlier that our comparative size—our smallness compared to the universe, or to anything else—cannot affect our value. It does not make things worthless, or worth less. Size doesn't matter.

Number can't affect intrinsic value either. But we saw earlier how number can affect significance: the more things there are to value, the less we can, and therefore ought, to, care about any one of them. And even if we set aside limits to our concern, it would still be the case that the more things there are to value, the less concern any one of them would receive, relative to the entire arc of concern. And number can also affect *extrinsic* value. It *can* make things worth more or less.

To see this, consider paradigm examples of extrinsic value. The remains of a shattered antique statue are more valuable if it is rare, if very few others like it exist. It will be even more precious if it is the only one of its kind.

This applies to us as well. If we are the only ones, then terrestrial sentience, and even more so, terrestrial intelligence, are utterly and incredibly rare and special. This would significantly increase our value. Indeed, although the vast emptiness that surrounds us does not in itself make an evaluative difference to our value and significance, it does, indirectly, greatly increase them. For arguably the larger and emptier the universe around us, the more special and rare we become. And the same would be all the more true if terrestrial life is but a brief blip of sentience after billions of years of darkness, to be succeeded by billions more when we go extinct.

In these ways, facts about the vast and indifferent cosmos we inhabit—some of the very same bleak facts that were supposed to make us insignificant—in fact increase our value, and thus also our significance. The vast, eternal silence that surrounds us may have frightened Pascal, but it is not entirely unwelcome.^43^

## Are We Valuable? Is Nothing Else Valuable?

The argument is embarrassingly simple. We possess value, and, if we are alone, nothing else in the universe does. Therefore we are the only thing that has value, and, trivially, possess most value. We're therefore of immense cosmic significance.

This argument assumes that we possess value. More precisely, it assumes that intelligence, and sentience more generally, confer value on the beings that possess them, and on their lives—and that we uniquely possess these attributes.

It does not matter, for our purposes here, what the source of this value is, or how exactly to spell it out. There are different accounts of how and why suffering is bad. There is more than one way to understand human flourishing, or the badness of death. But we need not resolve these disagreements here. It is enough that almost everyone agrees that we, and what happens to us, possess value in some respects. Opinions may vary about the value of our scientific or artistic achievements, and some might deny it outright. But it would be very hard, I believe, to find authors who sincerely deny that the prolonged agony and death of numerous innocent humans and other animals in no way matters—makes no difference to value. This would be sufficient for the first premise of the argument.^44^

Nihilists do, of course, deny even the badness of suffering, since they deny that anything matters. Since I have already discussed nihilism, we needn't return to it here. The question is whether we have substantive grounds for doubting that pain and death matter.

It is no doubt possible that we are mistaken about these matters. It's logically possible that there's nothing really bad about suffering. It is logically possible that ever growing emptiness is what really matters, so that in the distant future, when our universe slowly approaches a state of cold undifferentiated emptiness, things will actually be getting better and better… These are logical (if barely conceivable) possibilities, but to worry about them is to engage in idle scepticism. In the end, we cannot do better than to appeal to our deepest substantive convictions. And the conviction that suffering and sentient life matter commands overwhelming agreement.^45^

The claim that we possess value is admittedly ambiguous. It can refer to terrestrial sentient life in general, or it can refer only to us humans. If there is no life outside Earth, then life on Earth possesses unique value. Terrestrial life may be the only thing of value in the universe. But even if we humans are the only rational beings in the universe, it won't be true that we humans are the only thing of value. We're trivially *not* alone in *this* sense. We are surrounded by sentient life.

To claim that we humans possess cosmic significance, beyond the significance possessed by terrestrial life, we must further claim that our intelligence, and the achievements and failures it makes possible, are associated with a distinctive, superior kind of value. This judgment would still command a very broad agreement. Again, there are different ways in which it could be defended. Pascal singled out our capacity for thought, and Ramsey adds the capacity to love. Kant and others hold that rational agency is in itself of immense value; others would focus on morality. Even the minority who think that ultimately only sentience matters still almost invariably add that our intelligence, and attendant self-consciousness, mean that our capacity for well-being is far greater than that of other animals, and thus confers on us far more value.^46^ Despite these disagreements, these views all agree that we humans possess distinctive value here on Earth. If sentient life exists nowhere else in the universe, this means that we humans also possess a special cosmic significance.^47^

For the argument to support this conclusion about us humans, we need to add this further claim about our comparative terrestrial value, a claim that, as we saw, still commands wide agreement. But there is admittedly a small minority who denies that there is anything special about humanity. Some deny that our intelligence endows us with any distinctive value. Others go further and even deny the significance of sentience. They hold, for example, that what really matters is the terrestrial biosphere.^48^ This view would still imply that *the Earth* is of immense cosmic significance, if there is no life elsewhere in the universe. Would it also mean that we humans possess only derivative importance, as an undistinguished (and perhaps malignant) part of that larger whole? (That our significance is threatened, not by the immensity of the universe, but by the foliage around us…)

This doesn't follow. Even on this extreme view, we would possess considerable importance, even if it might take a less satisfying form. Even if our own value is undistinguished, what we humans do can affect the flourishing, even existence, of other terrestrial life in familiar ways. This would be enough to endow us with great importance, even if it is really the living Earth that ultimately matters.

There is very wide agreement that rationality and sentient life possess value. That was the first premise of the argument. Its second premise assumes that nothing else has final value. This second claim is also widely held. W. D. Ross, for example, wrote:

Contemplate any imaginary universe from which you suppose mind entirely absent, and you will fail to find anything in it you can call good in itself.^49^

Some would insist that we should also add living things to the list, even in the absence of sentience. Only few, I believe, would ascribe final value to what is neither itself rational, sentient or animate, nor dependent on these attributes.^50^ The early G. E. Moore, for example, held that natural beauty possesses some intrinsic value even when considered independently of its enjoyment by anyone.^51^ Others make similar claims about the value of sublime mountain ranges and majestic canyons. This does not yet threaten our terrestrial significance. Even the early Moore thought that, considered independently from any observer, the value of beauty is “so small as to be negligible”—that is, insignificant.^52^ And few if any think that if an asteroid attack created a magnificent series of craters, but in the course of doing so destroyed all life on Earth, then this would be a marked improvement.

However, if the Grand Canyon possesses final value, then presumably so do the Fra Mauro Formation on the moon, and the vast Valles Marineris on Mars—not to mention the probable trillions of exquisite canyons and mountains on innumerable unnamed alien planets. And if canyons possess final value, why not planetary systems, supernovas, or black holes? If we go this far, we might as well throw in the awe inspiring vast intergalactic darkness itself… So it seems that on this view, our universe, far from being utterly empty of value, in fact contains value nearly everywhere. Even if each instance of such value is negligible, it would still add up to an immensity when put together. And this immensity could swamp whatever value we terrestrials possess (and, if they exist, also the value of sentient beings elsewhere). Our significance, you might say, would be crushed by beauty…

This is an implausible result. If God had to choose between a barren universe filled with elaborate planetary chasms with no one to see them, and a topographically boring universe containing sentient life, it is clear which He should choose to create. It would be very peculiar, to put it mildly, to respond to the problem of evil by arguing that if we add up all the beauty in the universe, it would easily outweigh our puny suffering here on Earth.^53^ It would be even more absurd to peer at the night sky and feel utterly insignificant—because there are so many canyons out there!

To avoid these absurd conclusions, we must either deny that mountains and canyons and stars possess value except as objects of contemplation, or claim that even if they possess independent final value, this value is lexically inferior to the value possessed by rational, sentience beings. It is not necessary for my purposes to decide between the two. On the first, we'd be the only beings of value. On the second, we'd still have vastly more value than anything else, in a way that would justify giving us most attention and concern.^54^ To this we could add that if there really is all this *aesthetic* value out there, and we are the only ones capable of appreciating it, then this should surely only further amplify our significance.

The laws of nature are also sometimes claimed to possess final value. The universe, it is thought, would be worse if it didn't exhibit the order we see around us.^55^ Again, my argument doesn't require us to deny these claims. Even if the laws governing our universe are worthy of attention in their own right, it seems implausible that an observer of our universe should focus his interest on these laws and their multifarious manifestations, and treat sentient life as no more than an afterthought, or worse. Even if the laws governing our universe merit some attention—as, by the way, does its pervasive *emptiness*, and *lack* of value—this attention should soon shift to whatever glimmers of sentience and intelligence it contains, which may just be us. In fact, if the laws of nature possess value, one source of that value is precisely that they make life possible.

We would possess great cosmic significance if it is true that we possess value, that there's nothing else like us in the universe, and that nothing else possesses value. This is the simplest form of the argument. It faces the complication that *we humans* are not alone here on Earth. For the argument to establish that we humans are of great cosmic significance, we needed to add a premise about our value, and effect on value, compared to the rest of terrestrial sentient life, and life in general. In other words, we can possess great significance not only by being alone, but also by *standing out*. Further complications would be introduced if final value was also possessed by inanimate entities such as mountain ranges or the laws of nature. I argued that even if we admit such values, it is unlikely that this would threaten our cosmic significance. Just as we can be claimed to have great value compared to other terrestrial beings, we could also be claimed to have greater value compared to these other potential competitors.

## Does It Even Matter Whether We Exist?

It is common to write as if, seen from the cosmic standpoint, our existence doesn't matter, as if it makes no difference whether or not we exist. This is often presented as a consequence of the depressing fact that at some future point, the human species will die out. Russell lamented our future demise in “the vast death of the solar system”. In a letter from 1898, Joseph Conrad wrote that

The fate of a humanity condemned ultimately to perish… is not worth troubling about. If you take it to heart it becomes an unendurable tragedy. If you believe in improvement you must weep, for the attained perfection must end in cold, darkness and silence.^56^

The Christian apologist William Lane Craig writes more bluntly that, if God doesn't exist, then “[m]ankind is a doomed race in a dying universe. Because the human race will eventually cease to exist, it makes no ultimate difference whether it ever did exist.”^57^

Others accept our (species') mortality, and instead emphasize our inability to leave a lasting mark on the universe. Nicholas Rescher remarks that

on the astronomical scale, we are no more than obscure inhabitants of an obscure planet. Nothing we are or do in our tiny sphere of action within the universe's vast reaches of space and time makes any substantial difference in the long run.^58^

And Susan Wolf emphasizes our inability to “make a big and lasting splash,” or “a mark that will last forever”.^59^

But is it really true that our existence doesn't matter, that it makes no difference?

In Woody Allen's *Annie Hall*, a kid called Alvy complains that he is depressed. Asked why, he explains that the universe is expanding and “someday it will break apart and that would be the end of everything!” His disgusted mother replies ‘What is that *your* business?’ But there is a better reply.

Even the incurably optimistic have to accept that human life will eventually go extinct, perhaps sooner than later. If we exist longer, this will be better. But—to repeat an earlier point—whatever intrinsic value we have realized until then, and the intrinsic value of what we do now, cannot be affected by how long we go on existing.

Nor does our inevitable future demise threaten our significance. Thomas Nagel writes that “[f]rom outside, our birth seems accidental, our life pointless, our death unimportant, but from the inside, our life seems monstrously important and death catastrophic.”^60^ But, if we are considered collectively, as a species, then if we are alone, this is simply false. If we die out, intelligent life in the universe may have died out. And if we *hadn't* existed, nothing in the universe would have mattered.

These remarks also apply to the point that whatever we may achieve, as a species, will be erased by time, to the fact that we might not leave a lasting mark on the universe. It is common to see this as a threat, but this is again a mistake. This fact cannot affect whatever value we have realized, or erase our significance. To possess cosmic significance, we don't need to make any grand, lasting causal impact on the cosmic scale. Is the idea supposed to be that to be cosmically significant, we need to be moving galaxies around? But even a supernova, however immense in size or in its causal reach, is of no significance if it makes no difference to value. What might be true is that if we had greater causal powers, we would be able to make a greater difference in ways that *do* matter. In this way, we could be even more significant. But this point does not mean that we are not already very significant in the way I described.

We can therefore set aside worries about our future demise, and lack of lasting causal influence. And, as we have seen, if we're alone, and even more so if our existence is improbable, then it is also false to think that it simply does not *matter* whether we exist or not. If we don't exist, no one would, and things would be far darker, perhaps absolutely dark. We're not easily replaceable, to put it mildly, and our existence thus makes a *vast* difference.

There is another sense in which it is true that we are replaceable. If we're alone, then it matters greatly that we exist. But it's also true that it doesn't matter as much that *we* exist. It's good, from the point of view of the universe, if beings like us exist, but it's not especially important that these beings would be *us humans*, or anything especially like us. We just happen to be those who did emerge. Others could have emerged, whether here on Earth or elsewhere in the universe, who are as good, or much better. Nor are we tied in any special way to the unfolding cosmic events. Our existence is contingent, a kind of accident. But so what? Is that really a surprise, or cause for disappointment?

This is anyway a claim about what *might* have been. As things actually stand, now that we are here, we're not *at all* easily replaceable, if life is rare. Our extinction might be a momentous loss. Now that we exist, we do matter.

## Being in the Corner

The experience of cosmic insignificance is often blamed on the rise of modern science, and the decline of religious belief. Many think that things started to take a turn for the worse with Copernicus. Nietzsche, for example, laments ‘the nihilistic consequences of contemporary science’, and adds that

Since Copernicus it seems that man has found himself on a descending slope—he always rolls further and further away from his point of departure toward… —where is that? Towards nothingness?^61^

Freud later wrote about a series of harsh blows to our self-esteem delivered by science. The first blow was delivered by Copernicus, when we learned, as Freud puts it, that “our earth was not the centre of the universe but only a tiny fragment of a cosmic system of scarcely imaginable vastness…”^62^

It is still common to refer, in a disappointed tone, to the discovery that we aren't at the centre of God's creation, as we had long thought, but located, as Carl Sagan puts it, “in some forgotten corner”. We live, Sagan writes, “on a mote of dust circling a humdrum star in the remotest corner of an obscure galaxy.”^63^

But in what way is it a blow to discover that we are not in the centre of the universe? Why should it matter *where* we are located? (As if the universe is a massive theatre, and we have discovered that we got lousy seats.)

After all, believers don't think that *God* is located in the centre of the universe (indeed, if we are supposed to be in the centre, this won't leave God much room anyway…). Yet believers still take God to be pretty important. So being literally at the centre couldn't matter all that much, even in the enchanted world of the past. Actually, the traditional view was that hell is in the centre of the Earth, and in Dante's *Inferno*, at the very centre of the Earth, and thus of the universe, we find neither Man nor God, but Satan.^64^

In any event, in itself, spatial location matters no more than size does. Nor can it affect our intrinsic value. It would be even more ludicrous to tie our location in the universe to a denial that value exists, even that *objective* value exists. Our location has absolutely nothing to do with these metaethical claims.^65^

It might be replied that the worry is not about being literally at the centre, but about being central in the sense of being *important*.

But if God exists, then surely if anyone is in the centre, in this sense, it's obviously *God*, not us. If God exists, then trivially we *couldn't* be the most valuable entities in the universe, indeed we would be of absurdly negligible value compared to His perfect goodness—even if the sun and the moon revolved around us. Actually, we would probably be far lower down the ranks, for there may be angels, and numerous other wondrous beings in between. We would also, by the way, be far less significant than Satan.^66^

Many assume that if God doesn't exist, then we are doomed to insignificance.^67^ Theists who hold this view regard the naturalist alternative as unspeakably bleak. Atheists who hold it feel nostalgia to the enchanted world of religion.

This is a common view. But it gets things exactly backwards. The vastness and indifference of the universe present no challenge to our significance. But the existence of a perfect supreme being of infinite value certainly does.^68^

I do not mean to deny that the universe we inhabit is bleak, blind and indifferent. But our naturalistic universe nevertheless has some advantages. Contrary to what is commonly assumed, it is only if God *doesn't* exist, that we actually have a real shot at possessing focal cosmic significance—at being *genuinely central*.^69^

## If We Are Not Alone

Let us consider now what would follow, for our cosmic significance, if we are not alone. There are of course different ways in which this might be true, but I will assume, as seems plausible, that, if others exist, very many do.

The implications are obvious. We would lose much of our uniqueness, and thus much of the extrinsic value we would possess if we are alone. Our significance would be massively diluted.^70^ The cosmic spotlight would no longer be on us, and it would make little difference whether we exist, and whether we continue to exist. Our achievements, failures, and eventual extinction would no longer matter as much. We would be in no way central or especially important. And things would be worse if our greatest achievements pale in comparison to those of numerous more advanced civilizations.^71^

## Our Individual Significance

In other words, what threatens our significance is not at all the vastness of space, but whether there is anybody out there. It is thus false to simply assert that we are cosmically insignificant. We may well be, but we do not yet know. For all we now know, it may still turn out that we are the most important thing in the cosmos.

These, however, are claims about our *collective* cosmic significance, the significance of human beings. They are not even about the significance of currently living human beings, but of human beings throughout time, from the first ones till the future point when we will go extinct.

Our *individual* significance is a different matter. We saw that our collective significance depends on whether others exist. But, at the individual level, *we already know that others exist*: billions of other human beings, right here on little Earth. There are more than seven billion humans alive right now, and there have been many more in the past. And, unless we will go extinct soon, there will almost certainly be many more billions of humans in the future, probably swamping the number that exist now or have existed throughout history.

Our own individual persons seem to disappear in this vast crowd, and even if the cosmic spotlight is on us humans, it is most certainly not on any one of us. As individuals, we really are merely drops in this ocean.^72^

We already know, then, that we are terrestrially insignificant, even if humanity is cosmically significant. And things will be dramatically worse if we are not alone, and humanity turns out to be itself cosmically insignificant.^73^

In the second volume of *The Hitchhiker's Guide to the Galaxy*, Douglas Adams describes the ultimate method of execution. The ‘total perspective vortex’ is a contraption that presents its victims with a “one momentary glimpse of the entire unimaginable infinity of creation, and somewhere in it a tiny little mark, a microscopic dot on a microscopic dot, which says, ‘You are here’”.^74^ The apprehension of insignificance on this scale is supposed to be so shocking that the subject instantly dies.

The cosmic standpoint is not aligned with any metaethical position, but it does present a kind of objective measure of our significance—how we and our value rank overall. It is the ultimate seeing things in proportion. But to die from the shock of realizing just how small we are would be a sad mistake. Our status ‘in relation to the universe’ isn't determined by our size. It would rather be the glimpse of a pressing, never ending crowd of other persons, human and perhaps non-human (and, in an infinite or ‘many-worlds’ universe, also our numerous doppelgängers^75^), that should lead to the devastating vision of insignificance described by Adams.

If we feel insignificant as *individuals*, then this feeling is appropriate. But our insignificance is due to a terrestrial truism. It has little to do with nihilism or metaethics, or the vastness of the universe, or any other grand cosmological discovery.

To paraphrase Sartre, insignificance is other people.^76^

## Conclusion

The decline of religion, and the vast, indifferent universe revealed to us by natural science, have been a source of a distinctive kind of cosmic anxiety, a crushing sense that we are utterly insignificant. Such anxiety is by no means universal (not even in the embarrassingly parochial sense in which we use this term), but it is persistent.

But why on earth should we care about our cosmic significance? It might be thought that there is more than a touch of narcissism in this wish for cosmic celebrity, this desire to be, not just of some value, but better, or even the best.^77^ It should be unsurprising if such fantasies won't be fulfilled, and it would be childish to respond to this news with despair. We need to face it: we just aren't that special. Seen in this light, anxiety about our cosmic significance can begin to seem mildly embarrassing—it brings to mind madmen pretending to be Napoleon or Jesus.^78^

But such a verdict would be not only harsh, but also unfair. To begin with, concern about one's significance needn't take such a preposterous form. And most people wish, not to be of grand, God-like significance, but merely to *not* be *in*significant.^79^ Such a wish is still centered on the self, but I see little to criticize in it.^80^

Consider next that questions about significance, and cosmic significance, need not concern us, or our wishes. A concern with cosmic significance can be entirely outward looking. What is important deserves our attention. The cosmically important is what deserves our attention, when we adopt a viewpoint that encompasses everything. This needn't, of course, be us. If God exists, for example, He would be of immense cosmic significance. It goes without saying, that this is something that's important to know. Some religious traditions recommend a life of complete self-abnegation, devoted entirely to the humble contemplation of what is of cosmic importance—God, if He is there, the plight of sentient beings everywhere, the fundamental structure of reality… But even if we don't, or won't, adopt such an all encompassing viewpoint all of the time, it doesn't follow that we shouldn't adopt it at least some of the time. (We think it is important to know where life came from, how the universe begun, and how it will end. Surely, if this purely theoretical knowledge about the cosmos is important, then knowledge about what is cosmically important is *at least* as important.)

In any event, even if there is something distasteful in the *desire* for cosmic significance, it hardly follows that it doesn't matter whether we *are* cosmically significant. (That it's childish to fantasize about being a hero doesn't mean there are no heroes.) Those who mock concern about cosmic significance invariably also assume that we don't, and can't, have any. But would they still mock it if it turns out that we *are* cosmically important? (‘Turns out we are the most important thing in the universe—how boring, how *unimportant*!’)

There is something embarrassingly megalomaniac in the desire for grand cosmic significance. Even so, this desire might be fulfilled. It is highly unlikely that any of us possesses cosmic significance. But as is turns out, if we are alone in the universe then, taken together, we humans may nevertheless be of immense cosmic significance—and the irony is that we might have this significance precisely *because* the surrounding universe is so cold and indifferent.^81^

But there is little here that should flatter our ego. It would admittedly mean that we are the most important thing in the universe, singularly special and irreplaceable… But this won't be any kind of achievement, nothing we deserve credit for, or should be proud of. It's just an accident, an incredibly lucky accident. If we are the most significant, this isn't because we are so great. It is not so impressive to be the best when this just means being better than cosmic dust, or narrowly edging the apes. (And consider this: in a universe otherwise devoid of consciousness, even a glimmer of snail-like sentience would be precious.)

Nor could this significance satisfy any desire for cosmic celebrity or universal fame. If we possess great significance, we possess it precisely *because* there is no one else but us—and thus only when there is no one (but us) who can *appreciate* our significance. We may be centre stage, but the theatre is empty.

If anything, this should be sobering. It is not a cause for elation, but a burden, a great responsibility. If we are alone in the universe, the only thing of value, then this gives our continuing existence, and our efforts to avert disaster, a cosmic urgency, on top of whatever self-interested, anthropocentric reasons we have to stay around.^82^ That is to say, we might be *far more* important than we take ourselves to be. We humans are after all careless in numerous familiar ways, we fail to safeguard the future, or kick off pernicious habits. From a cosmic point of view, the problem wouldn't be that we suffer from an inflated sense of importance. It is that that we don't take our existence seriously enough.^83^

## Notes

1 Von Dokkum & Conroy, 2010.

2 Sagan, 1980.

3 Hawking said this in a 1995 interview.

4 Pascal, 2008, §205–206. The pessimist Leopardi echoes this passage in his ‘The Infinite’, 1819/1950; Paul Val*é*ry 1923/1971 later notoriously mocked it.

5 Conrad, 1914, 4; also cited in Nagel 1971.

6 Russell, 1903.

7 See John Donne, 1611/1966; Voltaire 1747/1978; Schopenhauer, 1851/2007, 19; Byron, letter of March 3, 1814/1904; Tolstoy, 2003, 820; Chesterton, 1908/2009, ch. 4; Camus, 1967; Nagel, 1971; 1986; 1987; Frankfurt, 1998, 304; Dworkin, 2000, 246; Wolf, 2007; 2010. See also Martin, 1992, 20–21; Weinberg, 2008. Stewart-Williams puts this most bluntly: “Science tells us that we are inconsequential specks of dust scrabbling around blindly on a pale blue dot orbiting a tiny star in an inconceivably large universe. Some say that size doesn't matter, but we all know that really it does. How can anything we do be important?” (2010, 189).

8 Nagel, 1971, 717.

9 Williams, 2008, 137. See also Frankfurt, 1998, 304; Blackburn, 2003. Even the few philosophers who take the worry seriously assume it must be metaethical in character. See, for example, Wiggins, 1976.

10 Mackie, 1991.

11 Williams singles out the cosmic angst in Russell, 1903 for special mockery. But that essay was written by the young, *Platonist* Russell. Far from expressing a worry about nihilism, Russell actually *presupposed* the existence of objective good. And the experience of insignificance is compatible even with theism, as demonstrated by Pascal and the author of *Ecclesiastes*.

12 Nagel presents a curious case in this regard. It is surprisingly unclear whether he sees the threat of insignificance as substantive or metaethical. In Nagel 1971, he appears to see it in metaethical terms, as the worry that our evaluative commitments are ultimately arbitrary and impossible to justify. This worry is later repeated in Nagel, 1987. But it's harder to interpret the discussion in the last chapter of Nagel 1986 in metaethical terms, and at one point Nagel explicitly rejects such a reading (1986, 219). Such a metaethical reading would be anyway inconsistent with his vigorous rejection, in an earlier chapter, of both nihilism and scepticism about value.

13 This actually follows from Moore's isolation test: we can tell what something's intrinsic value is by considering it in itself, as if it were *the only thing* in the universe. But then, by definition, it couldn't matter into that what kind of surrounding, large or small, we later place it back.

14 This point is implicit in Nagel, 1971. As McTaggart wrote, “if we do not start with the certainty that love for an hour on earth is unconditionally good, I do not see what ground we should have for believing that it would be good for an eternity in heaven.” (1909).

15 Korsgaard, 1983.

16 Russell, 1959. See also Nagel, 1971.

17 The word ‘significance’ is ambiguous in an unhelpful way, since it is associated both with importance and with *meaning* (things can *signify*, even *signify nothing*). I prefer to set aside questions about the meaning of life, or our cosmic meaning. These seem to me less clear, and perhaps less important. (For the distinction between meaning and importance, see Frankfurt, 1999).

18 Frankfurt 1999 claims, somewhat prematurely, that a non-circular analysis of ‘importance’ is impossible.

19 Miller, 1990, 40.

20 We can think of the relation between value and significance as parallel to that between having an absolute size and being large or small. If one is a certain size, then one just is that size. But how large or small one is depends on the comparison class: atoms, ants, rhinoceroses, quasars… And to know our comparative size relative to *everything else* is to know something we wouldn't know just by knowing our absolute size.

21 Worries about insignificance relate to substantive questions. They don't express metaethical worries about objectivity, realism or nihilism. They won't be made incoherent if antirealism is correct; nor are they settled simply by the truth of realism, in whatever strong form. But might our significance still be *affected* by the outcome of metaethical disputes? This depends on whether and how such disputes bear on substantive issues. This is a question I cannot enter into here.

22 The idea of a cosmic standpoint has also been unhelpfully associated, at least as far back as Sidgwick, with utilitarianism. But nothing about the cosmic standpoint, as described here, implies any kind of exclusive concern for the impersonal maximization of value. (Conversely, utilitarianism can do just fine without the cosmic standpoint; it merely requires the notion of the overall value of worlds.)

23 Notice however that although antirealism is *subject*-centric, it needn't be *anthro*pocentric. For example, antirealist views could ground value in what *all* rational beings will desire or agree to desire.

24 Williams, 2008, 137. To contrast, with Williams, the cosmic standpoint with the claim that value has its source in the human point of view is to trade on an ambiguity (‘anthropocentric’ shares in this ambiguity).

25 There is a minority that denies that we can intelligibly talk about the overall value of worlds and, presumably, of their parts. This minority might also have parallel doubts about the notion of overall significance, and thus also of unqualified talk of ‘absolute importance in the scheme of things’. Notice, however, that these conceptual doubts have nothing to do with the size of the universe or the objectivity of value. In fact, if they had force, they will have force *even if God exists*. This view implies that when God surveyed His creation on the sixth day, and declared it good, He was conceptually muddled.

26 The cosmic significance of things is their significance from an impartial, all encompassing standpoint. It isn't the same as their significance to us, or for us. Even if we are insignificant from that cosmic standpoint, we are no doubt very significant relative to our own, personal standpoint. Now it presumably isn't significant, from the cosmic standpoint, *whether* we (or whether *we*) are cosmically significant. The interesting question is whether our cosmic significance is significant *for* us—important from our personal (or collective human) perspective. I'll return to this question at the very end.

27 2003, 79.

28 Actually, the common metaphor comparing us to a speck of dust is already utterly inadequate—in relative terms, we are infinitesimally smaller than that. That metaphor actually expresses an inflated sense of self-importance.

29 Landau, 2011 makes a similar point.

30 The valueless does deserve our indifference. But this is not to say that, viewing things from the cosmic standpoint, we should stare intently at each patch of cosmic dust, and remain resolutely indifferent. Our indifference is better expressed by paying all of this vast expanse no attention—by turning our attention to what *does* have value.

31 See e.g. Lucretius, 1924; Bruno,1584/1998; Huygens, 1968/1968. Notice though that the expression ‘teeming with life’ is highly misleading. Even if there is life on billions of planets, it will still be the case that the universe is virtually utterly barren.

32 See Kant, 1996/1996, A825/B853.

33 As I write this, astronomers have for the first time identified a planet that appears to have the conditions for Earth-like life. Kepler 22-b is about 600 light years away, located well in the ‘habitable zone’ of the galaxy, and is broadly similar to the Earth in size and temperature.

34 Fermi's paradox is often taken to provide strong evidence for this. This argument was famously developed by Tipler, 1980.

35 *Pace* Wiggins, who writes that “[w]e have… abandoned the idea that the importance of emancipation or progress is that these are marks by which *our minute speck in the universe can distinguish itself as the spiritual focus of the cosmos*. Perhaps that is what makes the question of the meaning we can find in life so difficulty and so desolate for us…” (1976; my italics)

36 Wilde, 1892/2008.

37 But isn't our supposed cosmic significance merely a projection of familiar human concerns? We matter to ourselves, we disvalue pain, we find empty space, or orbiting gas giants, of no concern. It's thus not surprising that we turn out to be so special. But we win this competition only because we are the ones who set the rules. Realists can dismiss this worry. On their view, if sentience is intrinsically valuable, it's valuable whether or not we care about it, whether or not we exist. It's not as if value could have attached to anything in the universe, and it's somehow suspicious that it turns out to attach precisely to *us*—as if this is evidence of cosmic corruption. This objection has no force even if we accept antirealism. If we started out by worrying about our cosmic significance, and it turns out that we *are* significant, then this worry *has* been answered, even if value is ultimately grounded in our subjective concerns. And it's not as if antirealism *guarantees* our cosmic significance. Even if antirealism is true, this still depends on whether others exist.

38 Pascal, 2008.

39 Ramsey, 2000. Auden puts this somewhat differently: “Admirer as I think I am / Of stars that do not give a damn / I cannot, now I see them, say / I missed one terribly all day” (‘The More Loving One’, 1957/1976).

40 Notice that Pascal actually focuses, not simply on our rational capacities, but precisely on our capacity to adopt the *cosmic standpoint*—to form a view of everything.

41 I have found that Tegmark 2007 makes a similar point; Tegmark's views about the multiverse, however, are in tension with this conclusion, as we shall see below.

42 Ramsey, 2000. These two sentences precede the passage quoted earlier.

43 As Val*é*ry asserted, ‘The eternal silence of these infinite spaces *doesn't* frighten me’ (quoted in Huxley, 1931). Actually, the much lamented blindness and indifference of the universe also means that *our* sight, concern, and valuing are incredibly rare, and thus also more precious. As Wislawa Szymborska writes in her poem ‘The Ball’: ‘As long as nothing can be known for sure / (no signals have been picked up yet), / as long as Earth is still unlike / the nearer and more distant planets… / as long as our kindness / is still incomparable, / peerless even in its imperfection… / let's act like very special guests of honor… / and pretend that it's the ball / to end all balls.” (Szymborska, 2003).

44 Almost everyone agrees that sentient life possesses some value, even if pessimists like Schopenhauer, Leopardi and Zapffe think that this value is negative. A minority goes further, and ascribes final value to all living things, whether sentient or not (cf. Albert Schweitzer, 1966). To save words, I'll not always distinguish the value of sentient life from that of life itself. But below I'll briefly consider how some views about the value of life, and of intelligence, may affect my argument.

45 An objection: ‘Yes, *we* all agree that, say, suffering is awful. But do we really agree that suffering matters *from the point of view of the universe*?’ We mustn't take the cosmic standpoint too literally. We mustn't imagine an alien point of view, located somewhere in deep space, and which, for all we know, might find value in anything or nothing. Instead, we should begin with the assumption that some things possess value. Significance relates to what difference in value they make, compared to other things in view. Cosmic significance, to what difference they make, when *everything* is in view; it isn't the perspective of some peculiar being. As I said, the cosmic standpoint isn't the view from nowhere, but a view of everything.What we agree on is simply that suffering is awful. This is a claim about value, not about the cosmos or its perspective. But if suffering really is awful, then suffering would be just as awful when considered alongside the stars, or some extraterrestrial monument. To repeat: such a change in perspective cannot affect the intrinsic value of suffering, though it might bear on its significance. Some might still worry that, from a truly objective point of view, suffering isn't really bad. This might be an epistemic worry, the worry that even our deepest evaluative convictions are mistaken. But if they are mistaken, then pain isn't really bad, or significant, even on the parochial, *terrestrial* scale. If this epistemic worry is genuine, it arises even before we expand our scope to the cosmos. On another reading, the worry is not epistemic but metaethical. It's the worry that nothing is objectively bad in any strong sense—that nothing is bad in a sense that could be recognized even by an utterly alien standpoint. This metaethical worry has genuine force, and leads many to reject strong forms of realism. But it shouldn't lead them to reject my argument. Even if we agree that pain is bad, we may still disagree about the metaethics of value. But as I have argued earlier, questions about cosmic significance are neutral about this further disagreement.

46 It is worth also mentioning the contrary minority view: that *only* rational beings matter, and that other sentient beings matter only derivatively, in relation to rational beings. On this view, of course, our cosmic significance would be even greater, if no other rational beings exist.

47 To possess special significance, we humans merely need to have great value, and influence on value, compared to other terrestrial beings—though it would be simpler if our value was great*er* than that of all other terrestrial beings put together. What would happen, however, if the universe is full of sentient life, but we are the only rational beings? We might still possess distinctive value, but this value would be swamped by that of these innumerable alien life forms. To deny this result, we would need to further claim that intelligence and its associated benefits confer on us a value that is lexically superior to that of mere sentience. But since it seems unlikely that the universe is both teeming with sentient life *and* that there are no other rational beings, we needn't dwell on this possibility.

48 Cf. Arne N*æ*ss, 1973.

49 Ross, 1930, 140. See also Samuel Johnson, 1759/2009, 524; Sidgwick, 1784, I., ix, 4; III, xiv, 4–5; Russell, 1944/1951.

50 My argument is compatible, however, with holding that works of art, institutions, languages and so forth possess independent final value. These things wouldn't exist if intelligent beings don't exist.

51 Moore, 1903/1993, 135–136.

52 Ibid., 237–8. Moore later gave up even this claim. See his 1912, pp. 103–04, 148, 153.

53 Is this why God parades the wonders of the universe in His otherwise utterly unhelpful reply to the problem of evil in the book of *Job*?

54 As Melville 1852 writes: “That the starry vault shall surcharge the heart with all rapturous marvelings, is only because we ourselves are greater miracles, and superber trophies than all the stars in universal space”—and Melville presumably means: greater in value than all the stars in universal space, *put together*.

55 Ronald Dworkin defended something like this view in his recent Einstein Lectures in Berlin.

56 Frederick & Davies, 1986. For a contrary view, see Lenman, 2002.

57 2004, 290. Nietzsche 1976 similarly writes that, when it's all over for humanity, ‘nothing will have happened’, although unlike Craig, he finds this rather amusing (see also Nietzsche 1986, §49). Williams 2008 cites this passage approvingly, but omits the line where Nietzsche remarks on “how miserable, how shadowy and transient, how aimless and arbitrary the human intellect looks within nature.” This passage is far from denying our cosmic insignificance, or the intelligibility of worrying about it. It rather asserts it, in language not that far from Russell's. The difference is just that Nietzsche refuses to make a fuss about the whole thing.

58 1990, 153. See also James, 1907, lecture III. This is a recurring theme in the straw doggerel of professional pessimists.

59 Wolf, 2007.

60 Nagel, 1986, 209.

61 Nietzsche, 1967.

62 Freud, 1916, 284–5.

63 Sagan, 1980. Pascal had earlier described man as “lost in this remote corner of nature”. He adds: “…and from the little cell in which he finds himself lodged… let [man] estimate at their true value the earth, kingdoms, cities, and himself.” (2008, II, §72).

64 The common claim that Copernicus overturned the traditional anthropocentric picture of the universe is confused even as history. The Earth was thought to be in the centre—but this was often thought to be an appropriately *lowly* location. For example, Maimonides wrote that “…in the universe, *the nearer the parts are to the centre, the greater is their turbidness, their solidity, their inertness, their dimness and darkness*, because they are further away from the loftiest element…” and Aquinas thought that “in the universe, earth—that all the spheres encircle and that, as for place, lies in the center—is the most material and coarsest (*ignobilissima*) of all bodies.” For further discussion, see Danielson, 2001, as well as Blumenberg, 1987.

65 Actually, our location might bear on our significance, but *only* if God exists, since then our eccentric location might be evidence that we *aren't* significant to Him.

66 It might be objected that, on many theist views, God isn't *part* of the cosmos. This, however, is just a terminological point. The cosmic standpoint encompasses all existing entities. God is clearly one such entity, an entity of such supreme value that, according to theists, it should be the consuming focus of attention and concern. It is surely obvious that if God exists, He will possess paramount cosmic significance. (This is one reason why it's a mistake to identify the cosmic standpoint with God's point of view. From the cosmic point of view, God deserves much attention, but presumably God's attention and concern isn't largely focused on Himself! See n. 69 for further reasons to distinguish these two points of view.)

67 See e.g. Wolf, 2007.

68 This implication of theism has long been recognized. Isaiah writes that compared to God's gloriousness, “all our righteousnesses are as filthy rags.” (*Isaiah* 64:6 AV; see also *Isaiah* 40:15). And after being presented with the mysteries of the created cosmos, Job withdraws his complaint, exclaiming, in some translations, “Behold, I am insignificant; what can I reply to You?” (*Job* 40:4 NASU; the original goes ). Psalm 8 asks: “When I consider your heavens, the work of your fingers / the moon and the stars, which you have set in place / what is mankind that you are mindful of them / human beings that you care for them?” Such remarks pervade theism; see, for example, Locke, 1975, II, II, §3. Theists of course typically also hold that God cares about us *despite* our insignificance. You might wonder how *our* existence affects *God's* significance. Before He created us, and other sentient beings, God was alone: the only thing of value in the universe. But now we are also on the scene. Doesn't this diminish His significance, even by a little? On the other hand, we, and our value, are due to Him, so our addition should really *augment* God's significance. What this example shows is that significance couldn't be an entirely relational matter. See also n. 69.

69 (1) If God exists, we clearly won't be the most important thing, nor could we be of great importance, comparatively speaking. In a naturalist universe, if we are the only ones, we would be. These are claims about our *intra-world* significance. Theists could perhaps try to argue that we might nevertheless possess greater *cross-world* significance—that even if we'd deserve *most* attention *within* the naturalist universe, we'd nevertheless deserve *more* (or perhaps a *better* kind of) attention in a theist universe. It is enough, for my purposes, to have shown that the terms of the debate need to shift in this way. (2) Wouldn't God's concern *make* us cosmically significant, as Williams and others think? Notice first that cosmic significance refers to our importance, to what attention we deserve, not to what attention we actually get. God's concern may *respond* to our cosmic significance, but our significance isn't *identical* to being the object of such concern, nor does it require it. It's generally a mistake to identify the cosmic standpoint with God's point of view (or for Williams to *mock* it for this association), since God's point of view *needn't* be impartial—For example, He might care about us because we're His creations. And because of His supreme goodness, God might care about us *despite* the fact that we're insignificant. His concern might be *supererogatory*. (3) It might be replied that even if cosmic significance isn't the same as being the object of divine concern, such concern might still *endow* us with significance. If God has vast cosmic significance, and He cares about us, won't some of that significance rub off? This seems plausible. In a theist universe, God would deserve most notice. And we would deserve *more* notice because God notices us (as we notice some trees near Aix just because Cézanne noticed them—or as we notice a mote of dust on our beloved's forehead…). But this is merely to say that things would be *even worse* if *Deism* is true, and God couldn't care less. (On some theist views, God is the source of all value. This would mean that God's concern is also the source of *our* value. But this won't, by itself, establish our significance. As we saw earlier, value isn't sufficient for significance. For an illustration, see n. 71.)

70 Things would get monumentally worse, of course, if our universe is itself just a speck in a vast *multiverse*. Notice also that if we are not alone, Ramsey's picture would be false. Not because gas giants and nebulas would occupy centre stage—intelligent beings would still be in the foreground, it's just that it would get *extremely crowded*.

71 I've argued that if God exists, our cosmic significance would be negligible, compared to His. Some might still prefer being significant in this way, significant *to* God, than having most significance in a bleak Godless world. But notice that if others exist, this will dramatically further dilute our significance *even if God exists*. If others exist, then it's implausible that we are especially special to God, or play a key part in His divine plan. Theists have long recognized this threat, which is why some of them have been so resistant to the possibility that others exist—see e.g. William Whewell, 1853; Ellis, 1993, 394. Pat Robertson puts the point more crudely when he asserts that believers in ‘space aliens’ should be stoned… It might be replied as follows: ‘To be cosmically significant is to deserve cosmic attention. If God exists, He occupies the cosmic standpoint. But God has an unlimited attention span. If He cares about us, we receive cosmic attention. So how could we fail to be cosmically significant?’ This argument is flawed. As I pointed out earlier, it's a mistake to identify God's perspective with the cosmic standpoint (see ns. 66, 69). I also pointed out earlier that it's a mistake to think that we'd be literally *invisible* from the cosmic standpoint, if we possess some value. In this sense we're not literally of *zero* significance (see also ns. 73, 76). It doesn't follow, however, that we're *positively* significant—in any way important. For example, if others exist, we'd get far less of God's attention. His attention would be divided between this multitude. Not in the sense that we'd get less and less of a *fixed* amount, but that we'd get less and less of the *total* amount, even if we don't get less in absolute terms. In an obvious way, if Adam was the only intelligent being in the universe, then Adam would be immensely important (if also pretty lonely); he won't be remotely as important in a universe containing a sextillion other persons, even if he still received his fair share of attention.

72 By the way, on its own, immortality (in a crowded heaven or elsewhere) won't help with this—one of the main conceits of Mark Twain 1997. I'm grateful to Ritchie Robertson for this reference.

73 As individuals, we are cosmically *in*significant in the sense that we are *not* significant, in no way special or important. But, as we saw, it *doesn't* follow that we are *absolutely* insignificant, utterly invisible from the cosmic standpoint. Our number does make it to the vast cosmic phone book, if that's any consolation. To think otherwise is to overly anthropomorphize the cosmic standpoint: to think of it as constrained by contingent human limits. Actually, and ironically, we *might* also be absolutely insignificant—but *only* on antirealist views, since these often understand value, and thus significance (even on the cosmic scale) only by reference to human psychology and its limits.

74 Adams, 1980, 9.

75 If we inhabit a multiverse, there is even more emptiness than we had imagined. But what is most disturbing isn't this even greater emptiness, but the apparent implication, on certain variants of the view, that there exist innumerable doppelgängers who live all the possible lives we could have lived, and an even vaster horde of agents who realize every human (and non-human) possibility. In such a multiverse, it can seem as if it doesn't really matter what we do; some counterpart will anyway do the opposite. (The exact reverse of Nietzsche's eternal recurrence, where one choice, one path, is repeated *ad infinitum*.)

76 Some may still find it comforting to think that *all of us* are equally insignificant, that individual significance is simply out of reach. But this isn't obvious. It's possible that the variations in significance between us are ultimately tiny, not worth the effort. But they may be significant enough. It cannot be ruled out that at least a few of us possess non-trivial terrestrial significance and thus also, if we are alone, non-trivial cosmic significance. Think, for example, of someone who changes the course of history, even more so, someone who averts a genuine threat to the survival of humanity. It is true, however, that we aren't yet in a position to confidently estimate even the *terrestrial* significance of, say, Buddha, Napoleon or Hitler. As the Chinese premier Zhou Enlai once replied when asked about the significance of the French Revolution, it's still too early to say.

77 It might even have a sinister dimension. As Chesterton writes, “the joy of Satan in standing on a peak is not a joy in largeness, but a joy in beholding smallness, in the fact that all men look like insects at his feet.” He then suggests that “the evil of pride consists in being out of proportion to the universe.” (Chesterton, 1909).

78 To be fair, it's often really the true Napoleons of the world that manifest such grandiosity in its most extreme form. It is said that when Alexander was told that there is an infinite number of worlds, he responded in despair that he had not yet conquered even one… (Plutarch, 1939). It would be amusing if the desire for grand cosmic significance is ultimately just the projection of the evolved concern with status that we share with other primates…

79 In April 1944, Anne Frank wrote in her diary: “If God lets me live, I shall attain more than Mummy ever has done, I shall not remain insignificant, I shall work in the world for mankind.” God doesn't exist, and didn't let her live. But, in a cruel and twisted way, Anne Frank's wish for significance was fulfilled.

80 No doubt there are also those who would rather not disturb the universe. Some find the lightness of insignificance soothing—Leonard Woolf used to comfort himself with the thought that ‘nothing matters’ (Glenddining, 2006; see also McGinn, 1987, 272). But the question of our cosmic significance would be just as important on this view. It merely changes what answer we would hope to receive.

81 For further ironies of this sort, see the conclusion of Kahane, 2011.

82 See here DeLillo, 1983, in which two men in an aircraft orbiting the Earth wonder whether there is life elsewhere in the universe, while World War III rages below.

83 I am grateful to an anonymous reviewer for extremely helpful comments. I have also greatly benefited from comments by Alexandra Couto, Iddo Landau and Saul Smilansky, and from discussion with Nick Bostrom. Earlier versions of this paper were presented in Trinity College Dublin and Oxford, and at the *SPAWN* conference in Syracuse, where my commentator was Connie Rosati, to whom I am grateful. I learned much from many excellent—and otherwise motivating—suggestions in these events. The writing of this paper was supported by an award from the Wellcome Trust (WT087208MF).
